# Safety assessment of ripretinib: a real-world adverse event analysis from the food and drug administration adverse event reporting system

**DOI:** 10.3389/fonc.2025.1542315

**Published:** 2025-03-31

**Authors:** Sentai Wang, Hewen Chen, Yuying Zhou, Jianfeng Chen, Jiwei Cao

**Affiliations:** ^1^ Department of General Surgery, The Affiliated Suzhou Hospital of Nanjing Medical University, Suzhou Municipal Hospital, Gusu School, Nanjing Medical University, Suzhou, China; ^2^ Department of Neurosurgery, The Affiliated Suzhou Hospital of Nanjing Medical University, Suzhou Municipal Hospital, Gusu School, Nanjing Medical University, Suzhou, China; ^3^ Department of Thyroid Surgery, The Affiliated Suzhou Hospital of Nanjing Medical University, Suzhou Municipal Hospital, Gusu School, Nanjing Medical University, Suzhou, China

**Keywords:** ripretinib, FAERS, adverse event, disproportionality analysis, pharmacovigilance study

## Abstract

**Background:**

Ripretinib has been approved for the treatment of gastrointestinal stromal tumors (GIST). As a novel therapy, several adverse reactions remain unidentified, necessitating a thorough safety evaluation. This study analyzes real-world data from the US Food and Drug Administration Adverse Event Reporting System (FAERS) to investigate adverse events (AEs) associated with ripretinib.

**Methods:**

Adverse event reports (AERs) related to ripretinib were extracted from FAERS ASCII data spanning from the second quarter of 2020 to the second quarter of 2024. Following standardization, various disproportionality analyses, including the reporting odds ratio (ROR), proportional reporting ratio (PRR), bayesian confidence propagation neural network (BCPNN), and empirical bayes geometric mean (EBGM), were employed to identify potential safety signals linked to ripretinib. The data provided by medical professionals underwent sensitivity analysis to assess the robustness of the results.

**Results:**

A total of 3,105 ripretinib-related AERs were identified, categorized into 22 system organ classes (SOCs) and 84 preferred terms (PTs). Common AEs, such as alopecia, constipation, and muscle spasms, were consistent with the drug label and clinical trial findings. Notably, the risk of skin cancer associated with ripretinib was further elucidated. Additionally, new signals, including liver abscess and prostatomegaly, were detected. Despite their lower frequency, these signals demonstrated significant strength. A substantial proportion of adverse reactions (n = 322, 39.80%) occurred within the first month of treatment, although a smaller fraction emerged after one year. The sensitivity analysis revealed that most PTs related to skin and subcutaneous tissue maintained high signal values, with 8 cases of skin squamous cell carcinoma-related AEs still reported.

**Conclusion:**

The findings of this study align with established drug guidance and uncover new adverse event signals for ripretinib, thereby enhancing clinical monitoring and facilitating risk identification.

## 1 Introduction

Ripretinib is a novel oral type II tyrosine switch control inhibitor developed by Deciphera Pharmaceuticals. It specifically and durably inhibits the KIT proto-oncogene receptor tyrosine kinase (KIT) and the platelet-derived growth factor receptor A (PDGFRA), thus suppressing tumor cell growth (1). *In vitro* studies have demonstrated that ripretinib also inhibits various other kinases, including platelet-derived growth factor receptor β (PDGFRB), vascular endothelial growth factor receptor 2 (VEGFR2), angiopoietin-1 receptor (TIE2), and serine/threonine-protein kinase B-raf (BRAF) (2). In May 2020, ripretinib was approved in the United States as the first switch pocket-targeting TKI inhibitor for the treatment of advanced gastrointestinal stromal tumors (GIST) in adult patients who have previously received three or more kinase inhibitors (3). Clinical trial data indicate that ripretinib significantly extends both median progression-free survival and overall survival in patients with advanced GIST (4). Furthermore, ripretinib shows efficacy against other tumor types harboring KIT or PDGFRA mutations, including mastocytosis, leukemia, and lung cancer (1, 2). A clinical trial in metastatic melanoma reported an objective response rate of 23% (5). Additionally, ripretinib has exhibited encouraging synergistic effects in combination with other anti-tumor agents. For instance, the combination of ripretinib and the MEK inhibitor trametinib effectively targets GIST and systemic mastocytosis cells (6). Another study revealed that ripretinib combined with carboplatin significantly inhibits the proliferation of ovarian clear cell carcinoma (7).

Despite the substantial survival benefits of ripretinib for patients with advanced GIST, some individuals have had to pause or discontinue treatment due to severe adverse reactions, with certain events potentially being life-threatening (4). Therefore, early identification of adverse drug reactions and timely adjustments to therapeutic strategies are imperative. Clinical trials have identified common adverse events associated with ripretinib, including alopecia, actinic keratosis, muscle and joint pain, fatigue, nausea, decreased appetite, constipation, diarrhea, and palmar-plantar erythrodysesthesia syndrome (PPES) (5, 8, 9). As the number of patients treated with oral ripretinib increases, the reported adverse events have also risen. However, clinical trials, often constrained by sample size, may overlook some rare adverse reactions. In contrast, extensive real-world data can provide a more comprehensive understanding of ripretinib’s adverse drug reactions.

Pharmacovigilance refers to the long-term safety monitoring conducted after a drug’s market approval and involves a multifaceted process. This includes the collection of drug safety data, mandatory reporting of adverse reactions by pharmaceutical companies and healthcare professionals, solicitation of patient feedback regarding medication experiences, and the identification of potential safety signals that may indicate drug-related concerns (10). To date, many countries have established robust pharmacovigilance systems to monitor medication safety. These systems typically contain vast amounts of valuable real-world data, which are ideal for analysis using data mining algorithms in pharmacovigilance studies to detect potential safety signals associated with ripretinib. Spontaneous reporting systems remain the most widely utilized approach in pharmacovigilance, with the U.S. Food and Drug Administration Adverse Event Reporting System (FAERS) serving as the largest spontaneous reporting database for drug-related adverse events, accumulating over 20 million reports globally. No research has utilized the FAERS database to examine the adverse reactions associated with ripretinib to present date. Our objective is to evaluate the safety profile of ripretinib by analyzing extensive real-world patient adverse event data from FAERS, thereby offering valuable insights for clinical practice.

## 2 Methods

### 2.1 Data source

Data on adverse events (AEs) related to ripretinib were sourced from FAERS database (https://fis.fda.gov/extensions/FPD-QDE-FAERS/FPD-QDE-FAERS.html). The primary method of data collection in FAERS involves the voluntary submission of adverse reaction reports by healthcare professionals, consumers, and pharmaceutical manufacturers. For this study, ASCII report files were extracted from the FAERS database, covering the period from the second quarter of 2020 to the second quarter of 2024. The report files included seven distinct datasets: patient demographics (DEMO), drug (DRUG), reaction (REAC), outcome (OUTC), report source (RPSR), therapy (THER), and indications (INDI). In the FAERS database structure, these files are linked through unique identifiers such as PRIMARYID.

### 2.2 Data processing

**Figure 1:**
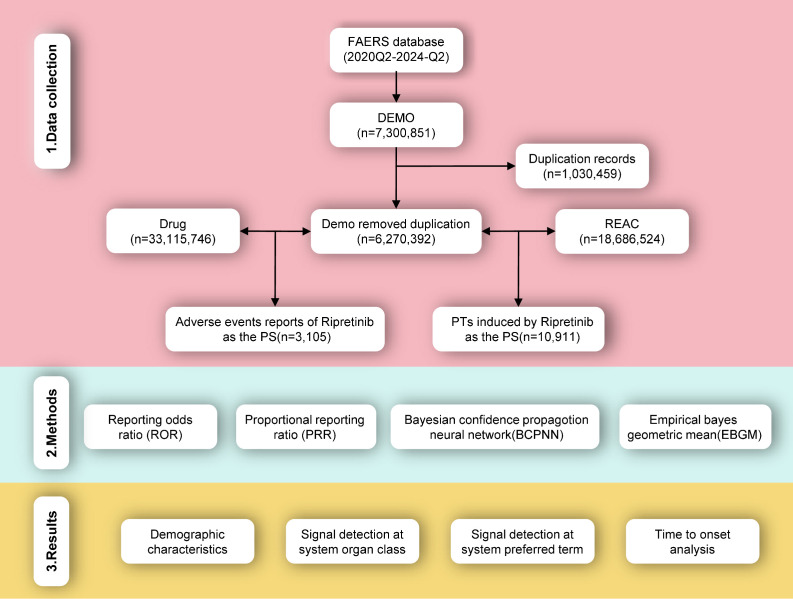
Flowchart of identifying AE cases of ripretinib from the FAERS database.

To ensure data accuracy, duplicate reports were removed in accordance with the Food and Drug Administration (FDA) recommendations. Data fields including PRIMARYID, CASEID, and FDA_DT, were extracted from the DEMO dataset and subsequently sorted. For reports sharing identical CASEID, the record with the most recent FDA_DT value was retained. In instances where both CASEID and FDA_DT values were identical, the record with the numerically largest PRIMARYID was preserved to ensure data uniqueness. When a patient experienced multiple AEs, each event was recorded separately and included in the analysis to ensure comprehensive safety signal detection. The drug role code in the event (ROLE_COD) is classified as primary suspect (PS), secondary suspect (SS), concomitant (C), or interaction (I). The Medex_UIMA_1.3.8 system was utilized to standardize drug names. Reports were filtered using “ripretinib” as a keyword in the DRUGNAME and PROD_AI columns, focusing specifically on cases where ripretinib was designated as the PS drug. The clinical characteristics of each report were collected, including demographic information (gender, age, reporter identity, reported country, and report year), drug indications, the date of AE occurrence, and its outcomes. If at least one of the following outcomes was reported, the event was classified as a serious AE: death, life-threatening, hospitalization, disability, or other serious events. The Medical Dictionary for Regulatory Activities (MedDRA 26.1) was employed to code the preferred terms (PTs) and system organ classes (SOCs) associated with ripretinib AEs. The specific screening procedure is illustrated in **Figure 1**.

### 2.3 Statistical analysis

Descriptive analysis was conducted to characterize all ripretinib-related AEs. Time to onset of ripretinib-related events was calculated by subtracting the administration date from the AE occurrence date. Disproportionality analysis techniques were employed, including the reporting odds ratio (ROR) (11), proportional reporting ratio (PRR) (12), bayesian confidence propagation neural network (BCPNN) (13), and empirical bayes geometric mean (EBGM) (14), to detect AEs. These four methods compare the incidence of AEs associated with the target drug to that of all other drugs. If this ratio exceeds a predefined threshold, it suggests disproportionality and may indicate a potential safety signal. ROR was the most widely employed disproportionality analysis method in pharmacovigilance due to its computational simplicity and ability to incorporate adjustments through logistic regression. Another significant advantage of ROR was its robustness against non-selective underreporting of either drugs or ADRs, which does not affect its calculated value (15). PRR quantified the ratio between observed and expected reporting frequencies (16), providing greater specificity than ROR (17). ROR and PRR, as frequency methods, exhibit high sensitivity but may yield false positives in instances of low AE counts (18). In contrast, BCPNN and EBGM, as Bayesian methods, were capable of effectively handling complex variables. BCPNN effectively mitigates stochastic errors in small-sample analyses while providing robust signal strength estimation through its Information Component (IC) metric (13). EBGM proved particularly valuable for analyzing multi-drug-event combinations, employing Bayesian shrinkage to suppress false-positive signals. Notably, EBGM estimates were considered more robust when the number of reports is limited (19–21). Each algorithm offered distinct advantages, and their combined use improved our ability to identify potential AEs more effectively. A PT was considered a positive signal when it met the threshold for all four algorithms simultaneously. All algorithms are based on 2×2 contingency tables, as detailed in **Supplementary Table S1**, with specific formulas and thresholds provided in **Supplementary Table S2**. Higher values indicate stronger signal strength, reflecting a more robust association between ripretinib and AEs. Statistical analyses were performed using R software version 4.4.0.

## 3 Results

### 3.1 Basic characteristics of AEs associated with ripretinib

From the second quarter of 2020 to the second quarter of 2024, a total of 6,270,392 AERs were collected from the FAERS database. Ripretinib was identified as the primary suspect in 10,911 cases of AEs, involving 3,105 patients. Reports from male patients were more frequent than those from females, comprising 54.40% of the total. The highest number of reports originated from the elderly group (over 65 years). Reports were primarily submitted by consumers (59.74%), followed by pharmacists (22.64%) and healthcare professionals (17.46%), with the majority originating from the United States (92.72%). A clear annual increase in reports was observed. Notably, severe outcomes such as hospitalization, death, life-threatening conditions, and disability accounted for nearly half of the total AERs. Additionally, our investigation revealed that beyond the FDA-approved use for GIST (71.92%), ripretinib has been utilized to treat other conditions, such as gastric cancer (0.75%). Detailed information is available in **Table 1**.

**Table 1:** Clinical characteristics of reports with ripretinib from the FAERS database.

Characteristics	Number of cases, n	Proportion, %
Gender		
Male	1689	54.40
Female	1349	43.45
Unknown	67	2.16
Age		
<18	1	0.03
18-65	530	17.07
>=65	787	25.35
Unknow	1787	57.55
Reporter		
Consumer	1855	59.74
Pharmacist	703	22.64
Physician	542	17.46
Unknown	5	0.16
Reported countries		
United States	2879	92.72
France	53	1.71
Canada	46	1.48
China	23	0.74
Others	104	3.35
Report year		
2020(Q2-Q4)	191	6.15
2021	686	22.09
2022	787	25.35
2023	900	28.99
2024(Q1-Q2)	541	17.42
Outcomes		
Hospitalization	567	38.18
Other serious	528	35.56
Death	371	24.98
Life threatening	16	1.08
Disability	3	0.20
Indications		
Gastrointestinal stromal tumour	2233	71.92
Gastric cancer	23	0.75
malignant melanoma	9	0.29
systemic mastocytosis	7	0.23
Others	833	26.81

### 3.2 Time-to-onset of ripretinib-associated AEs

**Figure 2:**
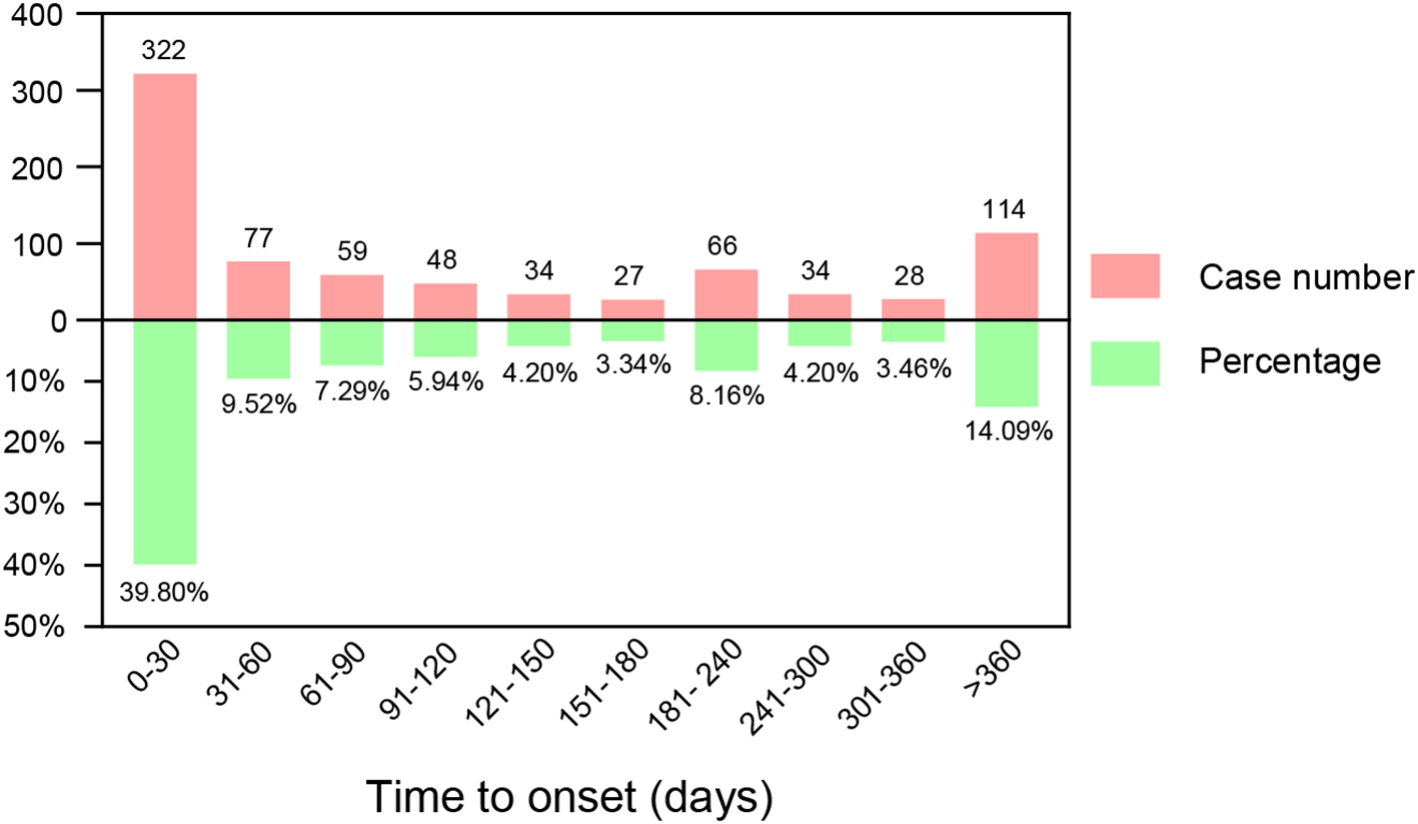
Time to onset of ripretinib-related adverse events (AEs).

After excluding reports with missing or incorrect onset times, 809 reported AEs met the inclusion criteria, with a median onset time of 62 days (interquartile range 8-220 days). The number of AEs over different time periods is depicted in **Figure 2**. Nearly 40% of AEs occurred within the first month of use. Although the number of AEs decreased over time, 14.09% of events could still occur more than a year after starting the medication.

### 3.3 Signal of SOCs

**Figure 3:**
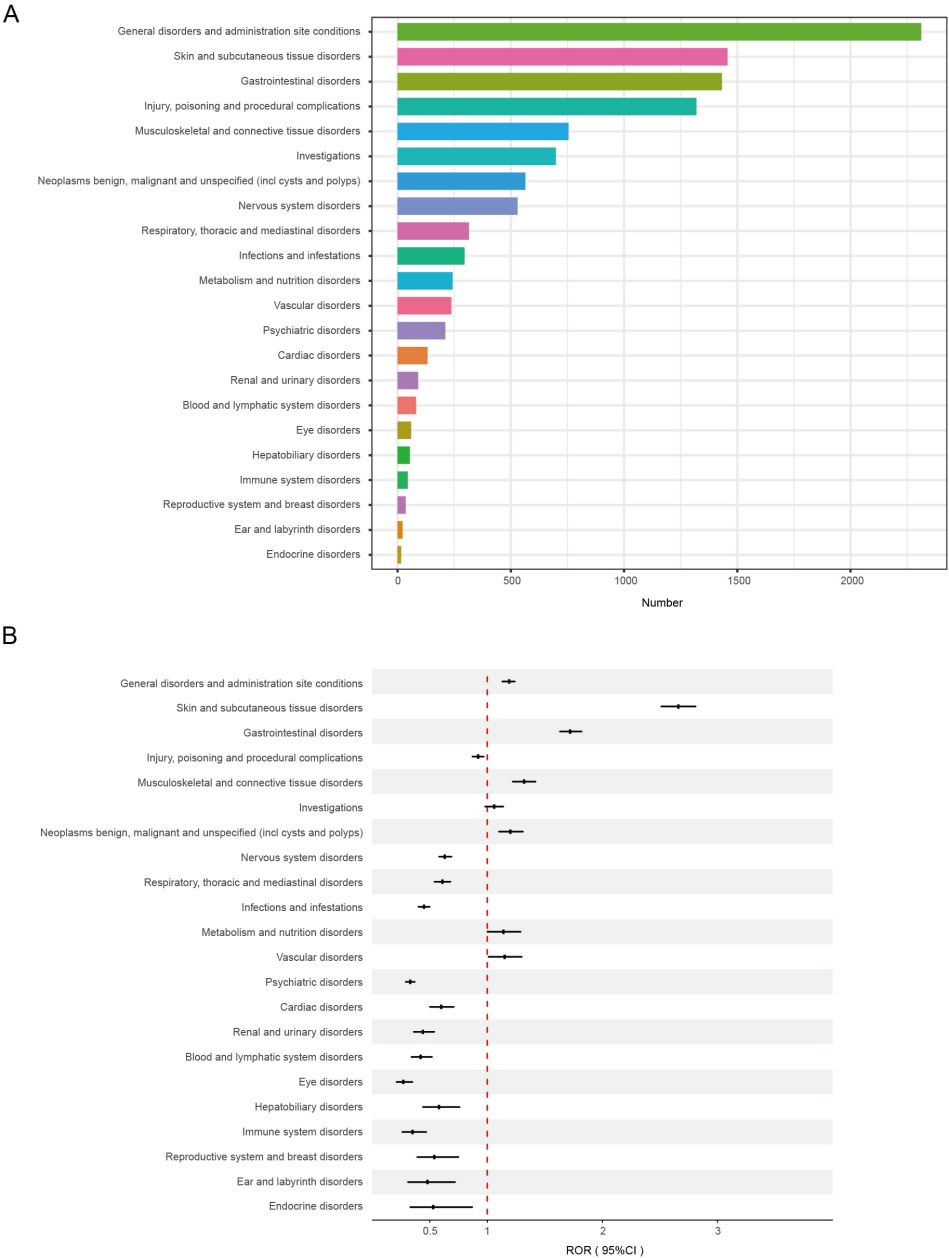
**(A)** The number of repretinib induced ADEs at the SOC level. **(B)** Signal detection at the SOC level.

A total of 22 SOCs were identified in this study, with the corresponding case numbers and ROR values presented in **Figure 3**. The four most prevalent systems were general disorders and administration site conditions (n = 2,312; ROR 1.19, PRR 1.15, IC 0.2, EBGM 1.15), skin and subcutaneous tissue disorders (n = 1,457; ROR 2.66, PRR 2.44, IC 1.28, EBGM 2.43), gastrointestinal disorders (n = 1,432; ROR 1.72, PRR 1.63, IC 0.7, EBGM 1.63), and injury, poisoning, and procedural complications (n = 1,320; ROR 0.92, PRR 0.93, IC -0.11, EBGM 0.93). Notably, skin and subcutaneous tissue disorders satisfied all four criteria. Detailed information is available in **Supplementary Table S3**.

### 3.4 Signal of PTs

**Figure 4:**
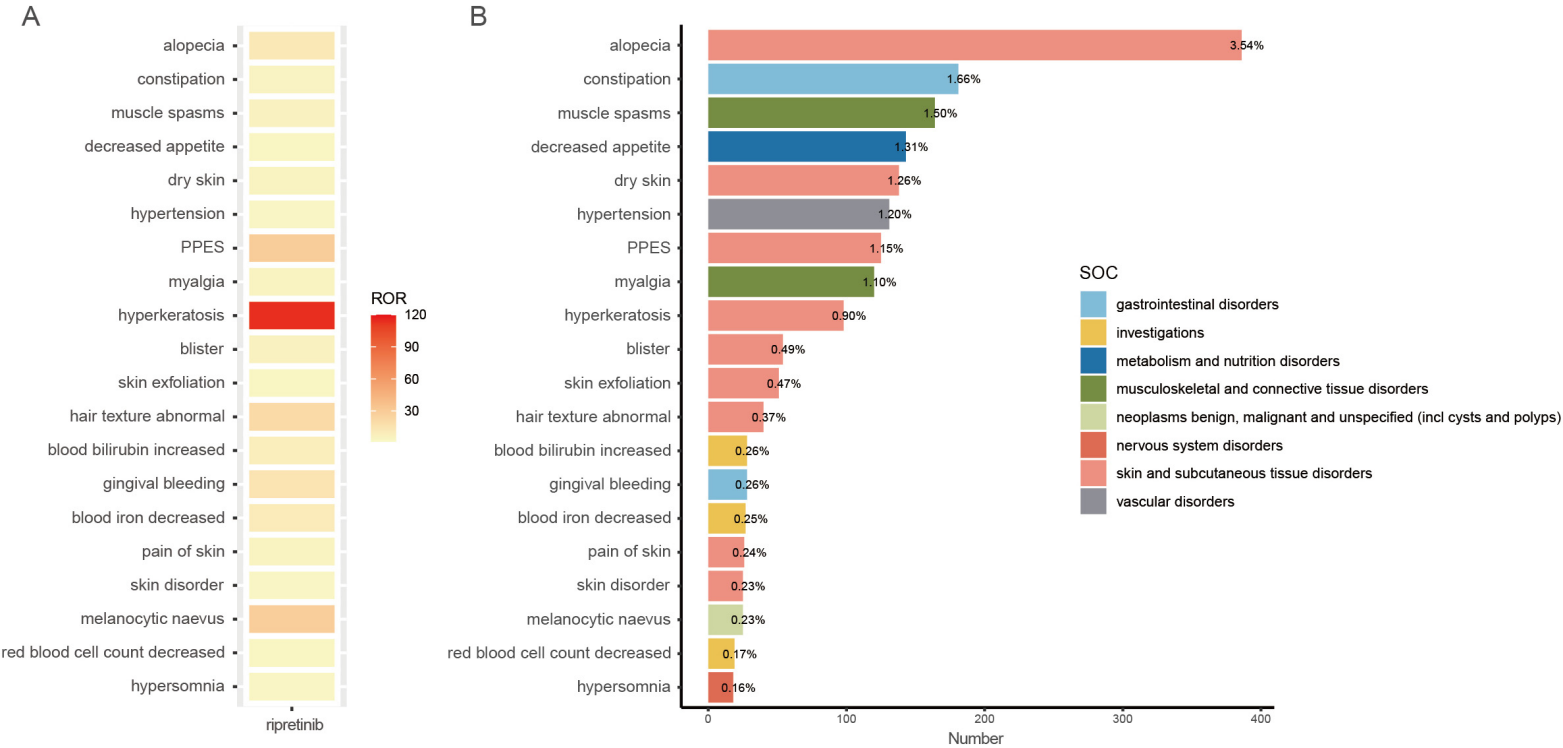
**(A)** Heatmap of the top 20 PTs ranked by numbers. **(B)** The number of PTs.

We identified 84 PTs that met all four screening criteria. The following three methods were used to exclude certain PTs: 1) Complications that may arise during the treatment of GIST; 2) PTs related to dosing errors or packaging issues; 3) PTs that did not specify the AE type. Detailed exclusion information were provided in **Supplementary Table S4**. The remaining PTs were sorted by case number, with the top 20 were shown in **Figure 4**. Among AEs reported more than 100 times, alopecia (n = 386), constipation (n = 181), muscle spasms (n = 164), decreased appetite (n = 143), dry skin (n = 138), hypertension (n = 131), PPES (n = 125), and myalgia (n = 120), all noted on the ripretinib drug label. The label also cites anemia, skin lesions (particularly certain malignancie), weight loss, paresthesia, oral discomfort, and specific laboratory abnormalities such as reduced blood phosphorus and calcium levels, which aligns with our findings. Interestingly, several common AEs listed on the drug label, including nausea, vomiting, fatigue, diarrhea, and heart failure, did not meet our four algorithm standards. Additionally, we observed AEs not documented on the label or in clinical trials, such as hypersomnia (n = 18; ROR = 3.7, PRR = 3.69, IC = 1.88, EBGM = 3.69), upper-airway cough syndrome (n = 7; ROR = 4.23, PRR = 4.23, IC = 2.08, EBGM = 4.22), increased tendency to bruise (n = 6; ROR = 4.25, PRR = 4.25, IC = 2.08, EBGM = 4.24), and prostatomegaly (n = 5; ROR = 7.5, PRR = 7.5, IC = 2.9, EBGM = 7.47).

To identify rare AEs, we ranked the EBGM signal strength, and **Table 2** presents the top 50 PTs. Results indicate that most of the top ten PTs with the strongest EBGM signals are associated with skin and subcutaneous tissue disorders. Notably, tongue hemorrhage (n = 3; EBGM = 4.24) and gingival bleeding also exhibited relatively high signal strength, suggesting an increased risk of bleeding associated with ripretinib. Notably, liver abscess (n = 3; EBGM = 5.45) was identified as the only infection-related PT that met the algorithm’s criteria. These results warrant careful attention from healthcare professionals.

**Table 2:** The top 50 AEs of ripretinib ranked by the EBGM at the PTs level.

SOC	Preferred term (PT)	Case numbers	ROR (95% two-side Cl)	PRR (95% two-side Cl)	IC (IC025)	EBGM (EBGM05)
skin and subcutaneous tissue disorders	Hyperkeratosis*	98	114.59 (93.32, 140.7)	113.57 (93.36, 138.16)	6.74 (6.44)	106.56 (89.74)
skin and subcutaneous tissue disorders	ephelides	8	84.07 (41.33, 170.99)	84.01 (41.49, 170.13)	6.32 (5.36)	80.12 (44.23)
reproductive system and breast disorders	nipple disorder	3	57.71 (18.26, 182.39)	57.7 (18.15, 183.4)	5.8 (4.36)	55.85 (21.32)
skin and subcutaneous tissue disorders	skin hypertrophy*	15	32.02 (19.2, 53.38)	31.97 (19.21, 53.22)	4.97 (4.26)	31.41 (20.48)
skin and subcutaneous tissue disorders	PPES*	125	29.64 (24.81, 35.41)	29.31 (24.57, 34.96)	4.85 (4.59)	28.84 (24.85)
neoplasms benign, malignant and unspecified (incl cysts and polyps)	melanocytic naevus	25	28.65 (19.29, 42.55)	28.58 (19.31, 42.3)	4.81 (4.25)	28.13 (20.2)
investigations	nutritional condition abnormal*	3	23.24 (7.44, 72.63)	23.23 (7.45, 72.4)	4.52 (3.09)	22.94 (8.84)
neoplasms benign, malignant and unspecified (incl cysts and polyps)	skin papilloma*	14	21.03 (12.41, 35.63)	21 (12.37, 35.65)	4.38 (3.64)	20.76 (13.35)
neoplasms benign, malignant and unspecified (incl cysts and polyps)	acrochordon	5	20.43 (8.46, 49.36)	20.43 (8.46, 49.35)	4.34 (3.17)	20.2 (9.66)
skin and subcutaneous tissue disorders	hair texture abnormal*	40	20.48 (14.99, 27.99)	20.41 (14.92, 27.93)	4.34 (3.89)	20.18 (15.54)
gastrointestinal disorders	tongue haemorrhage	3	18.41 (5.9, 57.44)	18.4 (5.9, 57.35)	4.19 (2.76)	18.22 (7.03)
gastrointestinal disorders	gingival bleeding*	28	14.79 (10.19, 21.46)	14.75 (10.16, 21.41)	3.87 (3.34)	14.63 (10.72)
investigations	blood chloride decreased	3	13.17 (4.23, 41.02)	13.17 (4.23, 41.05)	3.71 (2.29)	13.07 (5.05)
metabolism and nutrition disorders	weight gain poor*	5	12.63 (5.24, 30.44)	12.62 (5.22, 30.49)	3.65 (2.49)	12.54 (6)
skin and subcutaneous tissue disorders	alopecia*	386	11.8 (10.66, 13.07)	11.42 (10.35, 12.6)	3.5 (3.36)	11.35 (10.42)
investigations	blood electrolytes decreased	3	11.34 (3.64, 35.29)	11.34 (3.64, 35.34)	3.49 (2.07)	11.27 (4.36)
neoplasms benign, malignant and unspecified (incl cysts and polyps)	squamous cell carcinoma of skin*	12	10.55 (5.98, 18.61)	10.54 (5.97, 18.61)	3.39 (2.6)	10.48 (6.52)
investigations	blood iron decreased*	27	10.53 (7.21, 15.38)	10.51 (7.24, 15.25)	3.39 (2.85)	10.45 (7.61)
hepatobiliary disorders	hepatic lesion*	8	10.2 (5.09, 20.43)	10.19 (5.13, 20.23)	3.34 (2.4)	10.13 (5.66)
investigations	protein total decreased	4	8.68 (3.25, 23.19)	8.68 (3.26, 23.13)	3.11 (1.84)	8.64 (3.8)
skin and subcutaneous tissue disorders	hair growth abnormal	8	8.48 (4.23, 17)	8.48 (4.27, 16.84)	3.08 (2.13)	8.44 (4.72)
investigations	serum ferritin decreased	3	8.19 (2.63, 25.47)	8.19 (2.63, 25.53)	3.03 (1.61)	8.16 (3.16)
investigations	blood potassium abnormal	4	8.16 (3.06, 21.8)	8.16 (3.06, 21.74)	3.02 (1.75)	8.13 (3.57)
investigations	blood phosphorus decreased*	6	8.15 (3.65, 18.18)	8.14 (3.64, 18.18)	3.02 (1.95)	8.11 (4.14)
investigations	blood bilirubin increased*	28	8.1 (5.58, 11.74)	8.08 (5.57, 11.73)	3.01 (2.48)	8.04 (5.89)
reproductive system and breast disorders	prostatomegaly*	5	7.5 (3.12, 18.07)	7.5 (3.1, 18.12)	2.9 (1.74)	7.47 (3.58)
investigations	blood albumin decreased*	7	7.32 (3.48, 15.39)	7.32 (3.48, 15.42)	2.87 (1.86)	7.29 (3.92)
investigations	blood creatinine decreased*	4	6.73 (2.52, 17.96)	6.73 (2.53, 17.93)	2.74 (1.48)	6.7 (2.95)
gastrointestinal disorders	tongue ulceration	3	6.16 (1.98, 19.14)	6.16 (1.98, 19.2)	2.62 (1.2)	6.14 (2.38)
musculoskeletal and connective tissue disorders	muscle spasms*	164	6.04 (5.17, 7.04)	5.96 (5.1, 6.97)	2.57 (2.35)	5.94 (5.22)
skin and subcutaneous tissue disorders	hair colour changes*	12	5.7 (3.23, 10.05)	5.7 (3.23, 10.06)	2.51 (1.72)	5.68 (3.53)
skin and subcutaneous tissue disorders	skin atrophy	6	5.68 (2.55, 12.67)	5.68 (2.54, 12.69)	2.5 (1.43)	5.66 (2.9)
skin and subcutaneous tissue disorders	blister*	54	5.59 (4.28, 7.31)	5.57 (4.23, 7.33)	2.47 (2.09)	5.55 (4.44)
infections and infestations	liver abscess	3	5.46 (1.76, 16.97)	5.46 (1.75, 17.02)	2.45 (1.03)	5.45 (2.11)
investigations	vitamin b12 decreased	3	5.45 (1.75, 16.92)	5.45 (1.75, 16.99)	2.44 (1.02)	5.43 (2.1)
skin and subcutaneous tissue disorders	dry skin*	138	5.37 (4.54, 6.35)	5.31 (4.54, 6.21)	2.41 (2.16)	5.3 (4.61)
investigations	red blood cell count increased	3	5.29 (1.7, 16.45)	5.29 (1.7, 16.49)	2.4 (0.98)	5.28 (2.05)
skin and subcutaneous tissue disorders	pain of skin*	26	5.11 (3.47, 7.51)	5.1 (3.45, 7.55)	2.35 (1.8)	5.09 (3.68)
gastrointestinal disorders	tongue discomfort	5	4.98 (2.07, 11.99)	4.98 (2.06, 12.03)	2.31 (1.16)	4.97 (2.38)
musculoskeletal and connective tissue disorders	myalgia*	120	4.95 (4.13, 5.93)	4.91 (4.12, 5.86)	2.29 (2.03)	4.9 (4.21)
gastrointestinal disorders	constipation*	181	4.73 (4.08, 5.48)	4.67 (4.07, 5.36)	2.22 (2.01)	4.66 (4.12)
general disorders and administration site conditions	tenderness	8	4.47 (2.23, 8.95)	4.47 (2.25, 8.88)	2.16 (1.21)	4.46 (2.5)
gastrointestinal disorders	gingival pain	5	4.44 (1.85, 10.69)	4.44 (1.84, 10.73)	2.15 (0.99)	4.43 (2.13)
blood and lymphatic system disorders	increased tendency to bruise	6	4.25 (1.91, 9.48)	4.25 (1.9, 9.49)	2.08 (1.01)	4.24 (2.17)
respiratory, thoracic and mediastinal disorders	upper-airway cough syndrome	7	4.23 (2.02, 8.89)	4.23 (2.01, 8.91)	2.08 (1.08)	4.22 (2.27)
skin and subcutaneous tissue disorders	sensitive skin*	9	4.02 (2.09, 7.74)	4.02 (2.11, 7.68)	2.01 (1.11)	4.01 (2.32)
skin and subcutaneous tissue disorders	skin fissures	16	4.01 (2.46, 6.55)	4.01 (2.46, 6.55)	2 (1.31)	4 (2.65)
investigations	blood calcium decreased	7	3.78 (1.8, 7.94)	3.78 (1.79, 7.96)	1.92 (0.91)	3.77 (2.03)
musculoskeletal and connective tissue disorders	muscle atrophy*	7	3.76 (1.79, 7.89)	3.76 (1.79, 7.92)	1.91 (0.91)	3.75 (2.02)
skin and subcutaneous tissue disorders	skin disorder	25	3.74 (2.52, 5.54)	3.73 (2.52, 5.52)	1.9 (1.34)	3.73 (2.68)

* All four algorithms were still satisfied in the sensitivity analysis.

### 3.5 Sensitivity analysis

We selected AERs submitted by healthcare professionals for signal detection, identifying 1,245 case reports that documented 4,274 AEs. Analysis of the baseline characteristics revealed no significant differences in the distributions of gender, age, and the proportion of serious events (**Supplementary Table S5**). When evaluating the 22 SOCs, none met the criteria across all four algorithms. However, skin and subcutaneous tissue disorders yielded the highest values in all algorithms (**Supplementary Table S6**). For the PTs, three types were excluded based on a previously established methodology, leaving 42 PTs that met the criteria (**Supplementary Table S7**). Most PTs related to skin and subcutaneous tissue displayed high EBGM signal values, with 8 cases of cases of skin squamous cell carcinoma-related AEs still reported (ROR 13.14, PRR 13.12, IC 3.71, EBGM 13.05). After comparing with the PTs in **Table 2**, duplicate entries were marked with an asterisk in **Table 2**, indicating that these PTs demonstrated greater statistical robustness.

## 4 Discussion

This study utilized over four years of data from the FAERS database to comprehensively analyze AEs associated with ripretinib. The goal was to identify new significant risk signals through real-world data, thereby providing more comprehensive and accurate support for medical practice and public health decisions. Previously, most reports on ripretinib’s adverse reactions were derived from clinical trials, which often involved limited sample sizes that may have overlooked some rare but critical adverse reactions. A prior study using data from the European Spontaneous Adverse Event Reporting System analyzed ripretinib’s adverse reactions but, with only 176 cases, lacked the comprehensiveness needed for a thorough description (22). In contrast, our study collected a total of 10,911 ripretinib-related AEs involving 3,105 AERs, significantly exceeding the sample sizes of other studies. Based on the extensive dataset, we identified new AEs not previously recorded in drug labels or other studies, such as liver abscess and prostatomegaly.

Our baseline data indicated that the proportion of adverse reactions to ripretinib was slightly higher in males than in females, which may be related to the gender differences in the incidence of GIST (23). Reports were predominantly from the elderly group (aged over 65), consistent with epidemiological data indicating that the average onset age for GIST is 63. This trend may also be attributed to older adults being more susceptible to adverse drug reactions (24). Most adverse events occurred in the United States, likely due to ripretinib’s recent market entry and its limited approval or use in other countries. Additionally, aside from the currently approved indication for GIST, we noted some adverse reactions during treatment for other conditions like gastric cancer and mastocytosis. This off-label use may arise from challenges related to KIT inhibitor resistance caused by mutations in the drug target (25, 26), prompting some healthcare providers to utilize ripretinib for advanced patients. The increasing annual number of AERs reflects the gradual adoption of ripretinib, with serious outcomes accounting for nearly half of total AERs. This underscores the critical importance of monitoring ripretinib’s adverse reactions. Our time-to-onset analysis revealed irregularities in the timing of adverse reactions, highlighting the challenges faced in effectively monitoring these AEs.

Our disproportionality analysis identified 22 SOCs, with skin and subcutaneous tissue disorders being the most notable. Common reactions such as alopecia, dry skin, hyperkeratosis, and PPES align with the drug label. Alopecia was the most frequently reported AE, consistent with phase III clinical trial results (4), despite its rarity with other tyrosine kinase inhibitors (27). The mechanism behind ripretinib-related alopecia remains unclear but may involve the inhibition of kinases such as KIT, PDGFRA, VEGFR2, and BRAF, all of which have been linked to hair loss. Fortunately, most cases were graded as mild (grade 1, indicating < 50% hair loss) (28), and longitudinal analysis showed no progression over time (4). Dry skin was also a common adverse reaction, though its mechanism remains unknown. While epidermal growth factor receptor (EGFR) inhibitors can cause severe dryness (29), there is no evidence that ripretinib inhibits EGFR. Early clinical trials reported seborrheic keratosis and actinic keratosis with incidence rates exceeding 10%, but no severe reactions (grade 3 or higher) were observed, allowing for the continued use of the drug (9). PPES, a common adverse reaction associated with chemotherapy or targeted therapies, includes symptoms such as erythema, swelling, pain, and peeling on the palms and soles (30). Previous trials reported a high incidence of PPES, and similar to alopecia, most cases were mild and did not worsen over time (4). While the exact mechanism is unclear, it has been suggested that anti-angiogenic drugs may cause significant inflammation following vascular injury, particularly in high-pressure areas like the palms and soles (31). Other VEGFR inhibitors, such as sunitinib and sorafenib, have also reported multiple PPES reactions (32, 33).

In contrast to milder skin-related adverse reactions, primary skin malignancies represent a significant concern for ripretinib. In the INVICTUS trial, 4.7% of the 85 patients treated with ripretinib developed squamous cell carcinoma, while 2.4% developed melanoma (4). Our study recorded 12 cases of squamous cell carcinoma, along with 25 cases of melanocytic nevi, 14 papillomas, and 5 fibromas, indicating that ripretinib can induce various benign and malignant skin tumors (4). A review of patients who developed squamous cell carcinoma during ripretinib treatment showed an average age of 72, with lesions primarily in sun-exposed areas, displaying non-aggressive histopathological features similar to low-risk lesions induced by UV exposure (4). Therefore, patients on ripretinib, particularly the elderly, should be advised to avoid strong sunlight and undergo regular dermatological examinations. The mechanism by which ripretinib induces squamous cell carcinoma may relate to BRAF inhibition. A review summarized potential mechanisms by which BRAF inhibitors can induce squamous cell carcinoma, including individual HRAS gene mutations and infections with human papillomavirus (HPV) or human polyomavirus (HPyV) (34).

The increased susceptibility to HPVs seems to explain the reported cases of cutaneous papilloma in our results. Interestingly, studies have indicated that metastatic melanoma patients treated with BRAF inhibitors might develop new primary melanomas and atypical nevi, possibly linked to MAPK pathway activation (35). Although we did not report any cases of melanoma, we observed 25 cases of melanocytic nevi with high EBGM signal values. Previous research has demonstrated ripretinib’s potential for approval in treating advanced melanoma, and our data also reveal that a minority of melanoma patients have already undergone treatment with this drug, highlighting the need for careful monitoring of melanocytic lesions during the treatment period. Additionally, we identified previously unreported skin adverse reactions, such as freckles and skin thickening, with notably high EBGM signal values.

Other frequently reported AEs include constipation, appetite loss, hypertension, myalgia, muscle spasms, and decreased red blood cell counts, all of which align with findings from the package insert and clinical trials (4). Hypertension is a notable adverse reaction that may necessitate dosage reduction or temporary suspension of ripretinib, likely due to VEGFR2 inhibition (36, 37). In the INVICTUS study, 14% of GIST patients on ripretinib experienced grade 1-3 hypertension, with grade 3 hypertension constituting 7%, significantly higher than in the placebo group (4). Grade 2 hypertension requires a dose reduction, while grade 3 necessitates suspension of the drug. Therefore, managing blood pressure before initiating treatment and monitoring it throughout is vital. Although the incidence of anemia is relatively low in both clinical trials and our findings, its severity often leads to permanent discontinuation of treatment (4). However, the INVICTUS study indicated a higher incidence of anemia in the control group compared to the ripretinib group. Therefore, we attribute anemia more to systemic nutritional depletion from poorly controlled advanced gastrointestinal tumors rather than direct induction by ripretinib. Nonetheless, given its severity, enhancing nutritional support through iron supplementation or erythropoiesis-stimulating agents may be necessary during treatment. Another adverse reaction that requires attention is the risk of bleeding. We noted several bleeding-related AEs from FAERS database, including tongue bleeding, gum bleeding, tumor bleeding, and an increased tendency to bruise. The INVICTUS trial also reported laboratory coagulation abnormality such as increased activated partial thromboplastin time and international normalized ratio. This heightened bleeding risk may be linked to ripretinib’s VEGFR inhibition (2). A retrospective study suggested that combining low molecular weight heparin and other factor Xa inhibitors with VEGFR TKIs might further elevate the risk of bleeding (38). In contrast to the easily detectable and manageable oral bleeding, gastrointestinal bleeding from large tumors is often more insidious and life-threatening. This underscores the importance of closely monitoring coagulation function and exercising caution with other medications that may impact coagulation during ripretinib treatment.

Additionally, our analysis identified some unreported adverse reactions, such as liver abscess, upper-airway cough syndrome, and prostate hyperplasia. Although only three cases of liver abscess were reported, existing literature suggests that ripretinib may increase the risk of infections. The results of the INVICTUS study indicate that, compared to the control group, patients in the ripretinib group experienced a decrease in neutrophil counts, with one patient developing a perianal abscess (4). Currently, there is no clear evidence linking ripretinib directly to upper-airway cough syndrome or prostate hyperplasia, nor have other TKIs been reported to cause these issues. However, in the sensitivity analysis, prostatic hyperplasia still met the criteria of all four algorithms, suggesting the potential existence of undiscovered signaling pathways that may be sustaining this association. Further investigation is needed to clarify these relationships as ripretinib becomes more widely used.

Notably, many AEs reported were due to incorrect dosing, confusion over product appearance, and unclear packaging. These issues may stem from the relatively short time since ripretinib’s market introduction and its limited use, which contribute to unfamiliarity among healthcare professionals, and they should be preventable.

## 5 Limitations

While this study represents the first large-scale real-world investigation of adverse reactions to ripretinib, certain limitations must be acknowledged. The patients in the FAERS database are primarily from the United States, and the demographic data may not fully represent the broader population using ripretinib, potentially limiting the generalizability of the findings. Nearly 60% of the data originated from consumer spontaneous reports, which may introduce bias and lead to incomplete information. Severe adverse reactions might be underreported, as suggested by the absence of cardiac-related AEs noted in the package insert. Conversely, symptoms not caused by the drug may be overreported, potentially skewing the frequency and association of AEs and resulting in inaccurate conclusions. Additionally, due to the lack of comprehensive patient-level data, we are unable to assess the impact of confounding factors or conduct meaningful dose-response evaluations. Finally, signals of all AEs represent only statistical correlations, and further case-control studies and mechanistic research to establish causation are essential.

## 6 Conclusion

This study investigated the post-marketing safety characteristics of ripretinib using the FAERS database. Reports of AEs related to ripretinib were concentrated in 22 SOCs, and 84 positive signals were detected. It was found that skin-related adverse reactions are the most common and that ripretinib carries a risk of inducing skin cancer. The common AEs detected in this study are generally consistent with the manufacturer’s labeling and clinical trials. Additionally, the median time to onset of these AEs was analyzed, providing guidance for the safe clinical use of ripretinib.

## Data Availability

The raw data supporting the conclusions of this article will be made available by the authors, without undue reservation.

## References

[1] Schneeweiss M Peter B Bibi S Eisenwort G Smiljkovic D Blatt K. The kit and pdgfra switch-control inhibitor dcc-2618 blocks growth and survival of multiple neoplastic cell types in advanced mastocytosis. Haematologica. (2018) 103:799–809. doi: 10.3324/haematol.2017.179895 29439183 PMC5927976

[2] Smith BD Kaufman MD Lu WP Gupta A Leary CB Wise SC. Ripretinib (Dcc-2618) is a switch control kinase inhibitor of a broad spectrum of oncogenic and drug-resistant kit and pdgfra variants. Cancer Cell. (2019) 35:738–51 e9. doi: 10.1016/j.ccell.2019.04.006 31085175

[3] Kumar V Doros L Thompson M Mushti SL Charlab R Spehalski EI. Fda approval summary: ripretinib for advanced gastrointestinal stromal tumor. Clin Cancer Res. (2023) 29:2020–4. doi: 10.1158/1078-0432.CCR-22-2400 PMC10238554 36485007

[4] Blay JY Serrano C Heinrich MC Zalcberg J Bauer S Gelderblom H. Ripretinib in patients with advanced gastrointestinal stromal tumours (Invictus): A double-blind, randomised, placebo-controlled, phase 3 trial. Lancet Oncol. (2020) 21:923–34. doi: 10.1016/S1470-2045(20)30168-6 PMC8383051 32511981

[5] Janku F Bauer S Shoumariyeh K Jones RL Spreafico A Jennings J. Efficacy and safety of ripretinib in patients with kit-altered metastatic melanoma. ESMO Open. (2022) 7:100520. doi: 10.1016/j.esmoop.2022.100520 35753087 PMC9434165

[6] Gupta A Singh J Garcia-Valverde A Serrano C Flynn DL Smith BD. Ripretinib and mek inhibitors synergize to induce apoptosis in preclinical models of gist and systemic mastocytosis. Mol Cancer Ther. (2021) 20:1234–45. doi: 10.1158/1535-7163.MCT-20-0824 33947686

[7] Mori Y Okimoto Y Sakai H Kanda Y Ohata H Shiokawa D. Targeting pdgf signaling of cancer-associated fibroblasts blocks feedback activation of hif-1alpha and tumor progression of clear cell ovarian cancer. Cell Rep Med. (2024) 5:101532. doi: 10.1016/j.xcrm.2024.101532 38670097 PMC11149410

[8] Zhang X Zhang P Qiu H Fang Y Liu H Zhou Y. Large-scale, multicenter, prospective registry study of ripretinib in advanced gist: A real-world study from China. Adv Ther. (2023) 40:3817–29. doi: 10.1007/s12325-023-02576-0 PMC10427548 37356078

[9] Janku F Abdul Razak AR Chi P Heinrich MC von Mehren M Jones RL. Switch control inhibition of kit and pdgfra in patients with advanced gastrointestinal stromal tumor: A phase I study of ripretinib. J Clin Oncol. (2020) 38:3294–303. doi: 10.1200/JCO.20.00522 PMC7526717 32804590

[10] Pilipiec P Liwicki M Bota A. Using machine learning for pharmacovigilance: A systematic review. Pharmaceutics. (2022) 14:295–306. doi: 10.3390/pharmaceutics14020266 35213998 PMC8924891

[11] Rothman KJ Lanes S Sacks ST. The reporting odds ratio and its advantages over the proportional reporting ratio. Pharmacoepidemiol Drug Saf. (2004) 13:519–23. doi: 10.1002/pds.1001 15317031

[12] Evans SJ Waller PC Davis S. Use of proportional reporting ratios (Prrs) for signal generation from spontaneous adverse drug reaction reports. Pharmacoepidemiol Drug Saf. (2001) 10:483–6. doi: 10.1002/pds.677 11828828

[13] Bate A Lindquist M Edwards IR Olsson S Orre R Lansner A. A bayesian neural network method for adverse drug reaction signal generation. Eur J Clin Pharmacol. (1998) 54:315–21. doi: 10.1007/s002280050466 9696956

[14] Banks D Woo EJ Burwen DR Perucci P Braun MM Ball R. Comparing data mining methods on the vaers database. Pharmacoepidemiol Drug Saf. (2005) 14:601–9. doi: 10.1002/pds.1107 15954077

[15] van der Heijden PG van Puijenbroek EP van Buuren S van der Hofstede JW. On the assessment of adverse drug reactions from spontaneous reporting systems: the influence of under-reporting on odds ratios. Stat Med. (2002) 21:2027–44. doi: 10.1002/sim.1157 12111885

[16] Hauben M Reich L Chung S. Postmarketing surveillance of potentially fatal reactions to oncology drugs: potential utility of two signal-detection algorithms. Eur J Clin Pharmacol. (2004) 60:747–50. doi: 10.1007/s00228-004-0834-0 15619136

[17] Jiang Y Qu Y Du Z Ou M Shen Y Zhou Q. Exploring adverse events of vilazodone: evidence from the faers database. BMC Psychiatry. (2024) 24:371. doi: 10.1186/s12888-024-05813-0 38755677 PMC11100245

[18] Zou F Zhu C Lou S Cui Z Wang D Ou Y. A real-world pharmacovigilance study of mepolizumab in the fda adverse event reporting system (Faers) database. Front Pharmacol. (2023) 14:1320458. doi: 10.3389/fphar.2023.1320458 38186645 PMC10771301

[19] Kubota K Koide D Hirai T. Comparison of data mining methodologies using Japanese spontaneous reports. Pharmacoepidemiol Drug Saf. (2004) 13:387–94. doi: 10.1002/pds.964 15170768

[20] Hauben M Reich L. Safety related drug-labelling changes: findings from two data mining algorithms. Drug Saf. (2004) 27:735–44. doi: 10.2165/00002018-200427100-00004 15350157

[21] Almenoff JS LaCroix KK Yuen NA Fram D DuMouchel W. Comparative performance of two quantitative safety signalling methods: implications for use in a pharmacovigilance department. Drug Saf. (2006) 29:875–87. doi: 10.2165/00002018-200629100-00005 16970511

[22] Barbieri MA Sorbara EE Russo G Cicala G FranChina T Santarpia M. Neuropsychiatric adverse drug reactions with tyrosine kinase inhibitors in gastrointestinal stromal tumors: an analysis from the european spontaneous adverse event reporting system. Cancers (Basel). (2023) 15:1851. doi: 10.3390/cancers15061851 36980737 PMC10046586

[23] Tran T Davila JA El-Serag HB. The epidemiology of Malignant gastrointestinal stromal tumors: an analysis of 1,458 cases from 1992 to 2000. Am J Gastroenterol. (2005) 100:162–8. doi: 10.1111/j.1572-0241.2005.40709.x 15654796

[24] Stock S Redaelli M Simic D Siegel M Henschel F. Risk factors for the prescription of potentially inappropriate medication (Pim) in the elderly: an analysis of sickness fund routine claims data from Germany. Wien Klin Wochenschr. (2014) 126:604–12. doi: 10.1007/s00508-014-0589-2 25216754

[25] Wozniak A Gebreyohannes YK Debiec-Rychter M Schoffski P. New targets and therapies for gastrointestinal stromal tumors. Expert Rev Anticancer Ther. (2017) 17:1117–29. doi: 10.1080/14737140.2017.1400386 29110548

[26] Hornick JL Fletcher CD. The role of kit in the management of patients with gastrointestinal stromal tumors. Hum Pathol. (2007) 38:679–87. doi: 10.1016/j.humpath.2007.03.001 17437861

[27] Goggin C Stansfeld A Mahalingam P Thway K Smith MJ Huang P. Ripretinib in advanced gastrointestinal stromal tumors: an overview of current evidence and drug approval. Future Oncol. (2022) 18:2967–78. doi: 10.2217/fon-2022-0226 35880452

[28] Bauer S Jones RL Blay JY Gelderblom H George S Schoffski P. Ripretinib versus sunitinib in patients with advanced gastrointestinal stromal tumor after treatment with imatinib (Intrigue): A randomized, open-label, phase iii trial. J Clin Oncol. (2022) 40:3918–28. doi: 10.1200/JCO.22.00294 PMC9746771 35947817

[29] Lacouture ME Anadkat MJ Bensadoun RJ Bryce J Chan A Epstein JB. Clinical practice guidelines for the prevention and treatment of egfr inhibitor-associated dermatologic toxicities. Support Care Cancer. (2011) 19:1079–95. doi: 10.1007/s00520-011-1197-6 PMC3128700 21630130

[30] Banfield GK Crate ID Griffiths CL. Long-term sequelae of palmar-plantar erythrodysaesthesia syndrome secondary to 5-fluorouracil therapy. J R Soc Med. (1995) 88:356P–7P. PMC1295248 7629773

[31] Ishak RS Aad SA Kyei A Farhat FS. Cutaneous manifestations of anti-angiogenic therapy in oncology: review with focus on vegf inhibitors. Crit Rev Oncol Hematol. (2014) 90:152–64. doi: 10.1016/j.critrevonc.2013.11.007 24355408

[32] Xue Y Feng S Li G Zhang C. Safety profile of vascular endothelial growth factor receptor tyrosine-kinase inhibitors in pediatrics: A pharmacovigilance disproportionality analysis. Front Pharmacol. (2023) 14:1160117. doi: 10.3389/fphar.2023.1160117 37377925 PMC10291139

[33] Yamashita T Kudo M Ikeda K Izumi N Tateishi R Ikeda M. Reflect-a phase 3 trial comparing efficacy and safety of lenvatinib to sorafenib for the treatment of unresectable hepatocellular carcinoma: an analysis of Japanese subset. J Gastroenterol. (2020) 55:113–22. doi: 10.1007/s00535-019-01642-1 PMC6942573 31720835

[34] Wu JH Cohen DN Rady PL Tyring SK. Braf inhibitor-associated cutaneous squamous cell carcinoma: new mechanistic insight, emerging evidence for viral involvement and perspectives on clinical management. Br J Dermatol. (2017) 177:914–23. doi: 10.1111/bjd.15348 28129674

[35] Zimmer L Hillen U Livingstone E Lacouture ME Busam K Carvajal RD. Atypical melanocytic proliferations and new primary melanomas in patients with advanced melanoma undergoing selective braf inhibition. J Clin Oncol. (2012) 30:2375–83. doi: 10.1200/JCO.2011.41.1660 PMC3646308 22614973

[36] Camarda N Travers R Yang VK London C Jaffe IZ. Vegf receptor inhibitor-induced hypertension: emerging mechanisms and clinical implications. Curr Oncol Rep. (2022) 24:463–74. doi: 10.1007/s11912-022-01224-0 PMC9218917 35179707

[37] Versmissen J Mirabito Colafella KM Koolen SLW Danser AHJ. Vascular cardio-oncology: vascular endothelial growth factor inhibitors and hypertension. Cardiovasc Res. (2019) 115:904–14. doi: 10.1093/cvr/cvz022 30726882

[38] Patel SH George TL Wang TF Vogt SM Folefac E Xu M. Increased bleeding risk associated with concurrent vascular endothelial growth factor receptor tyrosine kinase inhibitors and low-molecular-weight heparin. Cancer. (2021) 127:938–45. doi: 10.1002/cncr.33337 33216354

