# Supplementary material for: Safety assessment of ripretinib: a real-world adverse event analysis from the food and drug administration adverse event reporting system
Source: Front Oncol. 2025 Mar 31;15:1542315. doi: 10.3389/fonc.2025.1542315 (PMC11994415; doi:10.3389/fonc.2025.1542315)
Supplement: Supplementary file 1 [file Table1.pdf]

Supplementary Material

Supplementary Table S1: four grid table.

	Ripretinib-related ADEs	Ripretinib-related ADEs	Total
Ripretinib	a	b	a + b
Non-Ripretinib	c	d	c + d
Total	a + c	b + d	N = a + b + c + d

Equation: a, number of reports containing both the target drug and the target adverse drug reaction; b, number of reports containing other adverse drug reactions of the target drug; c, number of reports containing the target adverse drug reaction of other drugs; d, number of reports containing other drugs and other adverse drug reactions; N, the number of reports.

Supplementary Table S2: ROR, PRR, BCPNN, and EBGM methods, formulas, and thresholds.

Method	Formula	Threshold
ROR	$\mathrm{ROR} = \frac{a/c}{b/d}$<br>$\mathrm{SE(lnROR)} = \sqrt{\frac{1}{a}+\frac{1}{b}+\frac{1}{c}+\frac{1}{d}}$<br>$95\%\mathrm{CI} = e^{\ln(\mathrm{ROR})\pm 1.96se}$	a ≥ 3<br>95%CI (lower limit) > 1
PRR	$\mathrm{PRR} = \frac{a/(a+b)}{c/(c+d)}$<br>$\mathrm{SE(lnPRR)} = \sqrt{\frac{1}{a}-\frac{1}{a+b}+\frac{1}{c}-\frac{1}{c+d}}$<br>$95\%\mathrm{CI} = e^{\ln(\mathrm{PRR})\pm 1.96se}$	a ≥ 3<br>PRR ≥ 2<br>95%CI (lower limit) > 1
BCPNN	$\mathrm{IC} = \log_2 \frac{p(x,y)}{p(x)p(y)} = \log_2 \frac{a(a+b+c+d)}{(a+b)(a+c)}$	IC025>0

$$E(IC) = \log_2 \frac{(a+\gamma 11)(a+b+c+d+\alpha)(a+b+c+d+\beta)}{(a+b+c+d+\gamma)(a+b+\alpha 1)(a+c+\beta 1)}$$

$$V(IC) = \frac{1}{(\ln 2)^2}\left[\frac{(a+b+c+d)-a+\gamma-\gamma 11}{(a+\gamma 11)(1+a+b+c+d+\gamma)} + \frac{(a+b+c+d)-(a+b)+a-\alpha 1}{(a+b+\alpha 1)(1+a+b+c+d+\alpha)} + \frac{(a+b+c+d+\alpha)-(a+c)+\beta-\beta 1}{(a+b+\beta 1)(1+a+b+c+d+\beta)}\right]$$

$$\gamma = \gamma 11 \frac{(a+b+c+d+\alpha)(a+b+c+d+\beta)}{(a+b+\alpha 1)(a+c+\beta 1)}$$

$$IC - 2SD = E(IC) - 2\sqrt{V(IC)}$$

$$EBGM = \frac{a(a+b+c+d)}{(a+c)(a+b)}$$

EBGM

$$SE(\ln EBGM) = \sqrt{\frac{1}{a}+\frac{1}{b}+\frac{1}{c}+\frac{1}{d}}$$

EBGM05>2

$$95\%CI = e^{\ln(EBGM)\pm 1.96se}$$

The EBGM, employs an empirical Bayesian approach, whereby a prior distribution is obtained by maximum likelihood estimates, and the prior and likelihood are subsequently combined to obtain a posterior distribution. The fifth percentile of the posterior distribution is denoted by “EBGM05” and is interpreted as the one-sided 95%confidence lower bound for the EBGM. Abbreviations: 95% CI, 95% confidence interval; IC, information component; IC025, the lower limit of the 95% CI, of the IC; E (IC), the IC, expectations; V (IC), the variance of IC; EBGM, empirical Bayesian geometric mean; EBGM05, empirical Bayesian geometric mean lower 95% CI, for the posterior distribution.

Supplementary Table S3: Signal strength of AEs of ripretinib at the System organ class (SOC) level in the FAERS database.

SOC	Case numbers	ROR (95% two-side Cl)	PRR (95% two-side Cl)	IC (IC025)	EBGM (EBGM05)
General disorders and administration site conditions	2312	1.19(1.13, 1.24)*	1.15(1.11, 1.2)	0.2(0.13)*	1.15(1.1)
Skin and subcutaneous tissue disorders	1457	2.66(2.51, 2.81)*	2.44(2.35, 2.54)*	1.28(1.2)*	2.43(2.32)*
Gastrointestinal disorders	1432	1.72(1.63, 1.82)*	1.63(1.57, 1.7)	0.7(0.62)*	1.63(1.55)
Injury, poisoning and procedural complications	1320	0.92(0.87, 0.97)	0.93(0.88, 0.99)	-0.11(-0.19)	0.93(0.89)

Musculoskeletal and connective tissue disorders	755	1.32(1.22, 1.42)*	1.29(1.19, 1.4)	0.37(0.27)*	1.29(1.22)
Investigations	699	1.06(0.98, 1.14)	1.05(0.97, 1.14)	0.08(-0.03)*	1.05(0.99)
Neoplasms benign, malignant and unspecified (incl cysts and polyps)	564	1.2(1.1, 1.31)*	1.19(1.1, 1.29)	0.25(0.13)*	1.19(1.11)
Nervous system disorders	530	0.63(0.58, 0.69)	0.65(0.6, 0.7)	-0.62(-0.75)	0.65(0.6)
Respiratory, thoracic and mediastinal disorders	315	0.61(0.54, 0.68)	0.62(0.55, 0.7)	-0.69(-0.85)	0.62(0.56)
Infections and infestations	296	0.45(0.4, 0.5)	0.46(0.41, 0.52)	-1.11(-1.28)	0.46(0.42)
Metabolism and nutrition disorders	243	1.14(1, 1.29)	1.13(1, 1.27)	0.18(0)*	1.13(1.02)
Vascular disorders	238	1.15(1.01, 1.3)*	1.14(1.01, 1.28)	0.19(0.01)*	1.14(1.03)
Psychiatric disorders	211	0.33(0.29, 0.37)	0.34(0.3, 0.39)	-1.56(-1.75)	0.34(0.3)
Cardiac disorders	132	0.6(0.5, 0.71)	0.6(0.5, 0.72)	-0.73(-0.98)	0.6(0.52)
Renal and urinary disorders	91	0.44(0.36, 0.54)	0.44(0.36, 0.54)	-1.18(-1.47)	0.44(0.37)
Blood and lymphatic system disorders	82	0.42(0.34, 0.52)	0.42(0.34, 0.52)	-1.25(-1.56)	0.42(0.35)
Eye disorders	60	0.27(0.21, 0.35)	0.28(0.22, 0.36)	-1.86(-2.22)	0.28(0.22)
Hepatobiliary disorders	54	0.58(0.44, 0.76)	0.58(0.44, 0.76)	-0.78(-1.17)	0.58(0.46)
Immune system disorders	45	0.35(0.26, 0.47)	0.35(0.26, 0.47)	-1.51(-1.93)	0.35(0.28)
Reproductive system and breast disorders	36	0.54(0.39, 0.75)	0.54(0.39, 0.75)	-0.88(-1.35)	0.54(0.41)
Ear and labyrinth disorders	22	0.48(0.31, 0.72)	0.48(0.32, 0.72)	-1.06(-1.65)	0.48(0.34)
Endocrine disorders	16	0.53(0.33, 0.87)	0.53(0.32, 0.87)	-0.91(-1.6)	0.53(0.35)

*Indicates statistically significant signals in the algorithm.

Supplementary Table S4: All PTs of ripretinib ranked by the ROR.

SOC	Preferred term (PT)	Case numbers	ROR (95% two-side Cl)	PRR (95% two-side Cl)	IC (IC025)	EBGM (EBGM05)
neoplasms benign, malignant and unspecified (incl cysts and polyps)	tumour compression*	10	192.49 (100.09, 370.2)	192.32 (100.72, 367.22)	7.43 (6.54)	172.99 (100.09)
skin and subcutaneous tissue disorders	hyperkeratosis	98	114.59 (93.32, 140.7)	113.57 (93.36, 138.16)	6.74 (6.44)	106.56 (89.74)
skin and subcutaneous tissue disorders	ephelides	8	84.07 (41.33, 170.99)	84.01 (41.49, 170.13)	6.32 (5.36)	80.12 (44.23)
neoplasms benign, malignant and unspecified (incl cysts and polyps)	tumour pain*	17	63.77 (39.28, 103.52)	63.67 (39.01, 103.93)	5.94 (5.26)	61.42 (40.95)

reproductive system and breast disorders	nipple disorder	3	57.71 (18.26, 182.39)	57.7 (18.15, 183.4)	5.8 (4.36)	55.85 (21.32)
neoplasms benign, malignant and unspecified (incl cysts and polyps)	gastric neoplasm*	5	46.79 (19.24, 113.78)	46.77 (19.36, 112.98)	5.51 (4.34)	45.55 (21.65)
injury, poisoning and procedural complications	extra dose administered**	305	39.15 (34.89, 43.92)	38.08 (33.86, 42.83)	5.22 (5.05)	37.28 (33.85)
investigations	scan abnormal	3	34.02 (10.85, 106.67)	34.01 (10.91, 106)	5.06 (3.63)	33.36 (12.82)
skin and subcutaneous tissue disorders	skin hypertrophy	15	32.02 (19.2, 53.38)	31.97 (19.21, 53.22)	4.97 (4.26)	31.41 (20.48)
skin and subcutaneous tissue disorders	PPES	125	29.64 (24.81, 35.41)	29.31 (24.57, 34.96)	4.85 (4.59)	28.84 (24.85)
neoplasms benign, malignant and unspecified (incl cysts and polyps)	hepatic neoplasm*	14	29.4 (17.33, 49.89)	29.37 (17.3, 49.86)	4.85 (4.12)	28.89 (18.56)
neoplasms benign, malignant and unspecified (incl cysts and polyps)	melanocytic naevus	25	28.65 (19.29, 42.55)	28.58 (19.31, 42.3)	4.81 (4.25)	28.13 (20.2)
neoplasms benign, malignant and unspecified (incl cysts and polyps)	abdominal neoplasm*	4	26.65 (9.92, 71.56)	26.64 (10, 70.98)	4.71 (3.44)	26.25 (11.49)
investigations	nutritional condition abnormal	3	23.24 (7.44, 72.63)	23.23 (7.45, 72.4)	4.52 (3.09)	22.94 (8.84)
neoplasms benign, malignant and unspecified (incl cysts and polyps)	cancer pain*	13	22.73 (13.15, 39.3)	22.71 (13.12, 39.31)	4.49 (3.73)	22.42 (14.18)
neoplasms benign, malignant and unspecified (incl cysts and polyps)	neoplasm progression*	213	22.69 (19.79, 26.01)	22.27 (19.41, 25.54)	4.46 (4.26)	21.99 (19.62)
neoplasms benign, malignant and unspecified (incl cysts and polyps)	skin papilloma	14	21.03 (12.41, 35.63)	21 (12.37, 35.65)	4.38 (3.64)	20.76 (13.35)
injury, poisoning and procedural complications	underdose**	240	21.01 (18.48, 23.9)	20.57 (18.29, 23.14)	4.35 (4.16)	20.34 (18.26)
skin and subcutaneous tissue disorders	hair texture abnormal	40	20.48 (14.99, 27.99)	20.41 (14.92, 27.93)	4.34 (3.89)	20.18 (15.54)
neoplasms benign, malignant and unspecified (incl cysts and polyps)	acrochordon	5	20.43 (8.46, 49.36)	20.43 (8.46, 49.35)	4.34 (3.17)	20.2 (9.66)
neoplasms benign,	spinal cord	4	20.38	20.38	4.33	20.15

malignant and unspecified (incl cysts and polyps)	neoplasm*		(7.6, 54.64)	(7.65, 54.3)	(3.06)	(8.83)
gastrointestinal disorders	tongue haemorrhage	3	18.41 (5.9, 57.44)	18.4 (5.9, 57.35)	4.19 (2.76)	18.22 (7.03)
neoplasms benign, malignant and unspecified (incl cysts and polyps)	metastases to peritoneum*	7	15.19 (7.22, 31.99)	15.19 (7.21, 31.99)	3.91 (2.91)	15.06 (8.08)
neoplasms benign, malignant and unspecified (incl cysts and polyps)	oncologic complication*	3	14.97 (4.8, 46.67)	14.97 (4.8, 46.66)	3.89 (2.47)	14.85 (5.74)
gastrointestinal disorders	gingival bleeding	28	14.79 (10.19, 21.46)	14.75 (10.16, 21.41)	3.87 (3.34)	14.63 (10.72)
investigations	computerised tomogram abnormal	4	14.48 (5.41, 38.75)	14.47 (5.43, 38.55)	3.84 (2.57)	14.36 (6.3)
general disorders and administration site conditions	adverse event***	180	13.99 (12.06, 16.22)	13.77 (12, 15.79)	3.77 (3.56)	13.67 (12.08)
neoplasms benign, malignant and unspecified (incl cysts and polyps)	neoplasm skin*	4	13.32 (4.98, 35.64)	13.32 (5, 35.49)	3.73 (2.45)	13.22 (5.81)
investigations	blood chloride decreased	3	13.17 (4.23, 41.02)	13.17 (4.23, 41.05)	3.71 (2.29)	13.07 (5.05)
neoplasms benign, malignant and unspecified (incl cysts and polyps)	gastrointestinal stromal tumour*	5	12.74 (5.28, 30.72)	12.74 (5.27, 30.78)	3.66 (2.5)	12.65 (6.06)
metabolism and nutrition disorders	weight gain poor	5	12.63 (5.24, 30.44)	12.62 (5.22, 30.49)	3.65 (2.49)	12.54 (6)
neoplasms benign, malignant and unspecified (incl cysts and polyps)	tumour haemorrhage*	6	12.35 (5.53, 27.57)	12.34 (5.52, 27.56)	3.62 (2.54)	12.26 (6.26)
skin and subcutaneous tissue disorders	alopecia	386	11.8 (10.66, 13.07)	11.42 (10.35, 12.6)	3.5 (3.36)	11.35 (10.42)
neoplasms benign, malignant and unspecified (incl cysts and polyps)	metastases to liver*	36	11.39 (8.2, 15.82)	11.36 (8.14, 15.85)	3.5 (3.03)	11.29 (8.58)
investigations	blood electrolytes decreased	3	11.34 (3.64, 35.29)	11.34 (3.64, 35.34)	3.49 (2.07)	11.27 (4.36)
neoplasms benign, malignant and unspecified (incl cysts and polyps)	squamous cell carcinoma of skin	12	10.55 (5.98, 18.61)	10.54 (5.97, 18.61)	3.39 (2.6)	10.48 (6.52)
investigations	blood iron decreased	27	10.53 (7.21, 15.38)	10.51 (7.24, 15.25)	3.39 (2.85)	10.45 (7.61)
hepatobiliary disorders	hepatic lesion	8	10.2 (5.09, 20.43)	10.19 (5.13, 20.23)	3.34 (2.4)	10.13 (5.66)
gastrointestinal	small intestinal	3	10.17	10.17	3.34	10.11

disorders	perforation*		(3.27, 31.65)	(3.26, 31.7)	(1.92)	(3.91)
general disorders and administration site conditions	disease progression*	237	10.1 (8.88, 11.49)	9.91 (8.81, 11.15)	3.3 (3.11)	9.85 (8.85)
investigations	protein total decreased	4	8.68 (3.25, 23.19)	8.68 (3.26, 23.13)	3.11 (1.84)	8.64 (3.8)
skin and subcutaneous tissue disorders	hair growth abnormal	8	8.48 (4.23, 17)	8.48 (4.27, 16.84)	3.08 (2.13)	8.44 (4.72)
investigations	serum ferritin decreased	3	8.19 (2.63, 25.47)	8.19 (2.63, 25.53)	3.03 (1.61)	8.16 (3.16)
investigations	blood potassium abnormal	4	8.16 (3.06, 21.8)	8.16 (3.06, 21.74)	3.02 (1.75)	8.13 (3.57)
investigations	blood phosphorus decreased	6	8.15 (3.65, 18.18)	8.14 (3.64, 18.18)	3.02 (1.95)	8.11 (4.14)
investigations	blood bilirubin increased	28	8.1 (5.58, 11.74)	8.08 (5.57, 11.73)	3.01 (2.48)	8.04 (5.89)
reproductive system and breast disorders	prostatomegaly	5	7.5 (3.12, 18.07)	7.5 (3.1, 18.12)	2.9 (1.74)	7.47 (3.58)
investigations	blood albumin decreased	7	7.32 (3.48, 15.39)	7.32 (3.48, 15.42)	2.87 (1.86)	7.29 (3.92)
gastrointestinal disorders	intra-abdominal fluid collection*	3	6.82 (2.19, 21.2)	6.82 (2.19, 21.26)	2.76 (1.35)	6.8 (2.63)
investigations	blood creatinine decreased	4	6.73 (2.52, 17.96)	6.73 (2.53, 17.93)	2.74 (1.48)	6.7 (2.95)
neoplasms benign, malignant and unspecified (incl cysts and polyps)	neoplasm*	15	6.63 (3.99, 11.01)	6.62 (3.98, 11.02)	2.72 (2.01)	6.6 (4.31)
gastrointestinal disorders	gastrointestinal perforation*	4	6.38 (2.39, 17.02)	6.37 (2.39, 16.97)	2.67 (1.4)	6.35 (2.79)
gastrointestinal disorders	tongue ulceration	3	6.16 (1.98, 19.14)	6.16 (1.98, 19.2)	2.62 (1.2)	6.14 (2.38)
musculoskeletal and connective tissue disorders	muscle spasms	164	6.04 (5.17, 7.04)	5.96 (5.1, 6.97)	2.57 (2.35)	5.94 (5.22)
skin and subcutaneous tissue disorders	hair colour changes	12	5.7 (3.23, 10.05)	5.7 (3.23, 10.06)	2.51 (1.72)	5.68 (3.53)
skin and subcutaneous tissue disorders	skin atrophy	6	5.68 (2.55, 12.67)	5.68 (2.54, 12.69)	2.5 (1.43)	5.66 (2.9)
skin and subcutaneous tissue disorders	blister	54	5.59 (4.28, 7.31)	5.57 (4.23, 7.33)	2.47 (2.09)	5.55 (4.44)
infections and infestations	liver abscess	3	5.46 (1.76, 16.97)	5.46 (1.75, 17.02)	2.45 (1.03)	5.45 (2.11)
investigations	vitamin b12 decreased	3	5.45 (1.75, 16.92)	5.45 (1.75, 16.99)	2.44 (1.02)	5.43 (2.1)
skin and subcutaneous tissue disorders	dry skin	138	5.37 (4.54, 6.35)	5.31 (4.54, 6.21)	2.41 (2.16)	5.3 (4.61)
investigations	red blood cell count increased	3	5.29 (1.7, 16.45)	5.29 (1.7, 16.49)	2.4 (0.98)	5.28 (2.05)

skin and subcutaneous tissue disorders	pain of skin	26	5.11 (3.47, 7.51)	5.1 (3.45, 7.55)	2.35 (1.8)	5.09 (3.68)
gastrointestinal disorders	tongue discomfort	5	4.98 (2.07, 11.99)	4.98 (2.06, 12.03)	2.31 (1.16)	4.97 (2.38)
musculoskeletal and connective tissue disorders	myalgia	120	4.95 (4.13, 5.93)	4.91 (4.12, 5.86)	2.29 (2.03)	4.9 (4.21)
gastrointestinal disorders	constipation	181	4.73 (4.08, 5.48)	4.67 (4.07, 5.36)	2.22 (2.01)	4.66 (4.12)
injury, poisoning and procedural complications	product dose omission in error**	40	4.67 (3.43, 6.38)	4.66 (3.41, 6.38)	2.22 (1.77)	4.65 (3.59)
general disorders and administration site conditions	tenderness	8	4.47 (2.23, 8.95)	4.47 (2.25, 8.88)	2.16 (1.21)	4.46 (2.5)
gastrointestinal disorders	gingival pain	5	4.44 (1.85, 10.69)	4.44 (1.84, 10.73)	2.15 (0.99)	4.43 (2.13)
injury, poisoning and procedural complications	product administration interrupted**	14	4.32 (2.55, 7.29)	4.31 (2.54, 7.32)	2.11 (1.37)	4.3 (2.77)
blood and lymphatic system disorders	increased tendency to bruise	6	4.25 (1.91, 9.48)	4.25 (1.9, 9.49)	2.08 (1.01)	4.24 (2.17)
respiratory, thoracic and mediastinal disorders	upper-airway cough syndrome	7	4.23 (2.02, 8.89)	4.23 (2.01, 8.91)	2.08 (1.08)	4.22 (2.27)
skin and subcutaneous tissue disorders	sensitive skin	9	4.02 (2.09, 7.74)	4.02 (2.11, 7.68)	2.01 (1.11)	4.01 (2.32)
skin and subcutaneous tissue disorders	skin fissures	16	4.01 (2.46, 6.55)	4.01 (2.46, 6.55)	2 (1.31)	4 (2.65)
investigations	blood calcium decreased	7	3.78 (1.8, 7.94)	3.78 (1.79, 7.96)	1.92 (0.91)	3.77 (2.03)
musculoskeletal and connective tissue disorders	muscle atrophy	7	3.76 (1.79, 7.89)	3.76 (1.79, 7.92)	1.91 (0.91)	3.75 (2.02)
skin and subcutaneous tissue disorders	skin disorder	25	3.74 (2.52, 5.54)	3.73 (2.52, 5.52)	1.9 (1.34)	3.73 (2.68)
nervous system disorders	hypersomnia	18	3.7 (2.33, 5.88)	3.69 (2.31, 5.91)	1.88 (1.23)	3.69 (2.5)
investigations	blood pressure abnormal	15	3.66 (2.2, 6.07)	3.65 (2.19, 6.08)	1.87 (1.16)	3.65 (2.39)
vascular disorders	hypertension	131	3.6 (3.03, 4.28)	3.57 (2.99, 4.26)	1.83 (1.59)	3.56 (3.08)
investigations	blood potassium decreased	17	3.53 (2.19, 5.68)	3.52 (2.2, 5.63)	1.81 (1.15)	3.52 (2.36)
metabolism and nutrition disorders	decreased appetite	143	3.44 (2.92, 4.06)	3.41 (2.92, 3.99)	1.77 (1.53)	3.4 (2.96)
investigations	red blood cell count decreased	19	3.41 (2.17, 5.35)	3.4 (2.17, 5.34)	1.77 (1.13)	3.4 (2.33)
injury, poisoning and procedural complications	prescribed underdose**	16	3.39 (2.08, 5.54)	3.39 (2.08, 5.53)	1.76 (1.07)	3.38 (2.24)
skin and subcutaneous	skin exfoliation	51	3.31	3.3	1.72	3.29

tissue disorders			(2.51, 4.36)	(2.51, 4.34)	(1.33)	(2.62)
investigations	laboratory test abnormal***	16	3.09 (1.89, 5.04)	3.08 (1.89, 5.03)	1.62 (0.94)	3.08 (2.04)

* Complications of malignant tumors
** Dosage or packaging issues
*** Not explicitly specified

Supplementary Table S5: Clinical characteristics of ripretinib reports submitted by medical professionals from the FAERS database.

Characteristics	Number of cases, n	Proportion, %
**Gender**		
Male	658	52.85
Female	532	42.73
Unknown	55	4.42
**Age**		
<18	0	0
18-65	215	17.27
>=65	349	28.03
Unknow	681	54.70
**Reporter**		
Pharmacist	703	56.47
Physician	542	43.53
**Reported countries**		
United States	1058	84.98
France	53	4.26
Others	134	10.76
**Report year**		
2020(Q2-Q4)	153	12.29
2021	442	35.5
2022	256	20.56
2023	255	20.48
2024(Q1-Q2)	139	11.16
**Outcomes**		
Hospitalization	265	35.67
Other serious	232	31.22
Death	227	30.55
Life threatening	16	2.15
Disability	3	0.40
**Indications**		
Gastrointestinal stromal tumour	853	68.51
Gastric cancer	9	0.72

malignant melanoma	6	0.48
systemic mastocytosis	6	0.48
Others	833	29.81

Supplementary Table S6: Signal strength of AEs of ripretinib at the System Organ Class (SOC) level in the sensitivity analysis.

SOC	Case numbers	ROR (95% two-side Cl)	PRR (95% two-side Cl)	IC (IC025)	EBGM (EBGM05)
general disorders and administration site conditions	1038	1.59(1.49, 1.71)*	1.45(1.37, 1.54)	0.54(0.44) *	1.45(1.37)
gastrointestinal disorders	562	1.71(1.56, 1.87) *	1.62(1.5, 1.75)	0.69(0.57) *	1.62(1.5)
injury, poisoning and procedural complications	535	1.03(0.94, 1.13)	1.03(0.95, 1.11)	0.04(-0.09)	1.03(0.95)
skin and subcutaneous tissue disorders	460	2.01(1.83, 2.22) *	1.91(1.77, 2.07)	0.93(0.79) *	1.9(1.76)
investigations	282	0.98(0.87, 1.11)	0.98(0.87, 1.1)	-0.02(-0.2)	0.98(0.89)
musculoskeletal and connective tissue disorders	263	1.22(1.07, 1.38) *	1.2(1.07, 1.35)	0.27(0.09) *	1.2(1.08)
nervous system disorders	213	0.67(0.58, 0.77) *	0.69(0.6, 0.79)	-0.54(-0.74)	0.69(0.61)
neoplasms benign, malignant and unspecified (incl cysts and polyps)	204	1.33(1.16, 1.53)	1.32(1.15, 1.51)	0.4(0.2) *	1.32(1.17)
respiratory, thoracic and mediastinal disorders	131	0.59(0.5, 0.71)	0.61(0.51, 0.73)	-0.72(-0.97)	0.61(0.52)
infections and infestations	103	0.33(0.27, 0.41)	0.35(0.29, 0.43)	-1.51(-1.8)	0.35(0.3)
metabolism and nutrition disorders	98	0.95(0.78, 1.16)	0.95(0.78, 1.16)	-0.08(-0.36)	0.95(0.8)
psychiatric disorders	76	0.43(0.35, 0.54)	0.44(0.35, 0.55)	-1.17(-1.5)	0.44(0.37)
vascular disorders	72	0.75(0.6, 0.95)	0.76(0.6, 0.96)	-0.4(-0.74)	0.76(0.62)
cardiac disorders	63	0.57(0.44, 0.73)	0.58(0.45, 0.75)	-0.8(-1.15)	0.58(0.47)
blood and lymphatic system disorders	45	0.36(0.27, 0.49)	0.37(0.28, 0.5)	-1.44(-1.85)	0.37(0.29)
renal and urinary disorders	44	0.52(0.39, 0.7)	0.53(0.39, 0.71)	-0.92(-1.35)	0.53(0.41)
hepatobiliary disorders	28	0.49(0.34, 0.71)	0.5(0.34, 0.73)	-1.01(-1.54)	0.5(0.36)
eye disorders	23	0.29(0.19, 0.44)	0.29(0.19, 0.44)	-1.76(-2.34)	0.29(0.21)
reproductive system and breast disorders	19	0.91(0.58, 1.43)	0.91(0.58, 1.43)	-0.14(-0.77)	0.91(0.62)
endocrine disorders	6	0.37(0.16, 0.82)	0.37(0.17, 0.83)	-1.44(-2.51)	0.37(0.19)
immune system disorders	5	0.08(0.03, 0.2)	0.08(0.03, 0.19)	-3.59(-4.74)	0.08(0.04)
ear and labyrinth disorders	4	0.29(0.11, 0.78)	0.29(0.11, 0.77)	-1.77(-3.03)	0.29(0.13)

*Indicates statistically significant signals in the algorithm.

Supplementary Table S7: All PTs of ripretinib ranked by number in the sensitivity analysis.

SOC	Preferred term (PT)	Case numbers	ROR (95% two-side Cl)	PRR (95% two-side Cl)	IC (IC025)	EBGM (EBGM05)
general disorders and administration site conditions	fatigue	178	3.88 (3.34, 4.51)	3.76 (3.28, 4.31)	1.91 (1.69)	3.76 (3.32)
skin and subcutaneous tissue disorders	alopecia	124	11.34 (9.48, 13.56)	11.04 (9.25, 13.17)	3.46 (3.2)	10.99 (9.46)
gastrointestinal disorders	constipation	66	5.2 (4.08, 6.63)	5.13 (4.05, 6.49)	2.36 (2.01)	5.12 (4.18)
musculoskeletal and connective tissue disorders	pain in extremity	46	3.07 (2.3, 4.11)	3.05 (2.27, 4.09)	1.61 (1.19)	3.05 (2.39)
skin and subcutaneous tissue disorders	palmar-plantar erythrodysaesthesia syndrome	44	18.96 (14.07, 25.55)	18.78 (14, 25.2)	4.22 (3.79)	18.62 (14.51)
musculoskeletal and connective tissue disorders	muscle spasms	44	4.93 (3.66, 6.63)	4.89 (3.64, 6.56)	2.29 (1.86)	4.88 (3.8)
musculoskeletal and connective tissue disorders	myalgia	41	4.39 (3.22, 5.97)	4.35 (3.18, 5.95)	2.12 (1.68)	4.35 (3.36)
gastrointestinal disorders	abdominal pain upper	34	3.34 (2.38, 4.68)	3.32 (2.38, 4.63)	1.73 (1.25)	3.31 (2.5)
skin and subcutaneous tissue disorders	dry skin	34	4.71 (3.36, 6.61)	4.68 (3.35, 6.53)	2.22 (1.74)	4.67 (3.52)
skin and subcutaneous tissue disorders	hyperkeratosis	25	87.16 (58.37, 130.15)	86.66 (58.56, 128.25)	6.38 (5.81)	83.36 (59.61)
gastrointestinal disorders	abdominal distension	19	3.47 (2.21, 5.45)	3.46 (2.2, 5.43)	1.79 (1.15)	3.45 (2.37)
skin and subcutaneous tissue disorders	skin exfoliation	17	3.48 (2.16, 5.6)	3.47 (2.17, 5.55)	1.79 (1.12)	3.46 (2.32)
investigations	blood bilirubin increased	15	7.15 (4.3, 11.89)	7.13 (4.28, 11.87)	2.83 (2.12)	7.11 (4.65)
skin and subcutaneous tissue disorders	blister	14	3.54 (2.09, 5.98)	3.53 (2.08, 5.99)	1.82 (1.09)	3.52 (2.27)
skin and subcutaneous tissue disorders	hair texture abnormal	13	74.73 (42.96, 130.01)	74.51 (43.04, 128.99)	6.17 (5.4)	72.06 (45.34)
musculoskeletal and connective tissue disorders	bone pain	10	3.56 (1.91, 6.62)	3.55 (1.9, 6.65)	1.83 (0.97)	3.55 (2.11)
skin and subcutaneous tissue disorders	acne	8	3.64 (1.82, 7.29)	3.63 (1.83, 7.21)	1.86 (0.92)	3.63 (2.03)
neoplasms benign, malignant and unspecified (incl cysts and polyps)	squamous cell carcinoma of skin	8	13.14 (6.55, 26.35)	13.12 (6.61, 26.05)	3.71 (2.76)	13.05 (7.29)
investigations	blood iron decreased	7	8.94 (4.25, 18.79)	8.93 (4.24, 18.81)	3.15 (2.15)	8.89 (4.78)
skin and subcutaneous tissue disorders	pain of skin	7	7.53 (3.58, 15.82)	7.52 (3.57, 15.84)	2.91 (1.9)	7.5 (4.03)
investigations	blood	7	4.4	4.4	2.13	4.39

	potassium decreased		(2.1, 9.25)	(2.09, 9.27)	(1.13)	(2.36)
investigations	blood sodium decreased	5	5.39 (2.24, 12.96)	5.38 (2.23, 13)	2.42 (1.27)	5.37 (2.58)
neoplasms benign, malignant and unspecified (incl cysts and polyps)	skin papilloma	5	16.79 (6.96, 40.49)	16.77 (6.94, 40.51)	4.06 (2.9)	16.65 (7.97)
skin and subcutaneous tissue disorders	skin irritation	5	4.49 (1.87, 10.81)	4.49 (1.86, 10.85)	2.16 (1.01)	4.48 (2.15)
gastrointestinal disorders	eructation	4	7.67 (2.87, 20.48)	7.66 (2.87, 20.41)	2.93 (1.67)	7.64 (3.36)
investigations	blood phosphorus decreased	4	9.88 (3.7, 26.39)	9.87 (3.7, 26.3)	3.3 (2.03)	9.83 (4.32)
metabolism and nutrition disorders	weight gain poor	4	30.65 (11.42, 82.27)	30.62 (11.49, 81.59)	4.92 (3.64)	30.21 (13.22)
gastrointestinal disorders	gingival bleeding	4	5.65 (2.12, 15.07)	5.64 (2.12, 15.03)	2.49 (1.23)	5.63 (2.48)
reproductive system and breast disorders	prostatomegaly	4	20.55 (7.67, 55.04)	20.54 (7.71, 54.73)	4.35 (3.07)	20.35 (8.93)
skin and subcutaneous tissue disorders	hair colour changes	4	13.72 (5.13, 36.7)	13.71 (5.15, 36.53)	3.77 (2.5)	13.63 (5.99)
hepatobiliary disorders	hepatic lesion	4	10.2 (3.82, 27.26)	10.2 (3.83, 27.18)	3.34 (2.07)	10.15 (4.46)
skin and subcutaneous tissue disorders	skin hypertrophy	4	23.51 (8.77, 62.99)	23.48 (8.81, 62.56)	4.54 (3.27)	23.24 (10.19)
investigations	blood albumin decreased	4	7.25 (2.71, 19.35)	7.24 (2.72, 19.29)	2.85 (1.58)	7.22 (3.17)
nervous system disorders	hyperaesthesia	3	6.3 (2.03, 19.56)	6.29 (2.02, 19.6)	2.65 (1.23)	6.28 (2.43)
respiratory, thoracic and mediastinal disorders	hiccups	3	6.34 (2.04, 19.69)	6.33 (2.03, 19.73)	2.66 (1.24)	6.32 (2.45)
investigations	nutritional condition abnormal	3	48.54 (15.45, 152.46)	48.5 (15.56, 151.16)	5.57 (4.13)	47.46 (18.21)
investigations	blood thyroid stimulating hormone increased	3	5.58 (1.8, 17.34)	5.58 (1.79, 17.39)	2.48 (1.06)	5.57 (2.16)
metabolism and nutrition disorders	appetite disorder	3	7.99 (2.57, 24.83)	7.98 (2.56, 24.87)	2.99 (1.57)	7.96 (3.08)
skin and subcutaneous tissue disorders	sensitive skin	3	9.1 (2.93, 28.28)	9.09 (2.92, 28.33)	3.18 (1.76)	9.06 (3.51)
musculoskeletal and connective tissue disorders	muscle atrophy	3	6.37 (2.05, 19.79)	6.37 (2.04, 19.85)	2.67 (1.25)	6.35 (2.46)
investigations	blood creatinine decreased	3	10.21 (3.28, 31.75)	10.2 (3.27, 31.79)	3.34 (1.93)	10.16 (3.93)
general disorders and administration site conditions	sluggishness	3	7.96 (2.56, 24.74)	7.96 (2.55, 24.81)	2.99 (1.57)	7.93 (3.07)
